# A Simple Microbiological Tool to Evaluate the Effect of Environmental Health Interventions on Hand Contamination

**DOI:** 10.3390/ijerph111111846

**Published:** 2014-11-17

**Authors:** Carol Devamani, Guy Norman, Wolf-Peter Schmidt

**Affiliations:** 1 Department of Disease Control, Faculty of Infectious and Tropical Diseases, London School of Hygiene and Tropical Medicine, Keppel Street, London WC1E 7HT, UK; E-Mail: wolf-peter.schmidt@lshtm.ac.uk; 2 Water and Sanitation for the Urban Poor, Capital Tower, 91 Waterloo Road, London SE1 8RT, UK; E-Mail: gnorman@wsup.com

**Keywords:** sanitation, hygiene, contamination

## Abstract

The effects of interventions such as sanitation or hand hygiene on hand contamination are difficult to evaluate. We explored the ability of a simple microbiological test to: (1) detect recontamination after handwashing; (2) reflect risk factors for microbial contamination and (3) be applicable to large populations. The study was done in rural Andhra Pradesh, India, and Maputo, Mozambique. Participants placed all 10 fingertips on a chromogenic agar that stains *Enterococcus* spp. and *E. coli* spp. Outcomes were the number of colonies and the number of fingertips with colonies. In the recontamination study, participants were randomised to handwashing with soap and no handwashing, and tested at 30 min intervals afterwards. In two cross sectional studies, risk factors for hand contamination were explored. Recontamination of hands after washing with soap was fast, with baseline levels reached after 1 h. Child care was associated with higher *Enterococcus* spp. counts, whereas agricultural activities increased *E. coli* spp. counts. Food preparation was associated with higher counts for both organisms. In Maputo, counts were not strongly associated with water access, latrine type, education or diarrhoea. The method seems unsuitable for the evaluation of handwashing promotion. It may reflect immediately preceding risk practices but not household-level risk factors.

## 1. Introduction

A considerable proportion of the transmission of gastro-intestinal pathogens is thought to occur via hands [1,2]. Environmental interventions such as improved sanitation, water access or hand washing are thought to decrease the risk of hand contamination and consequently the risk of food contamination and direct contact transmission [3,4]. Despite application of various epidemiological methods, the effect of environmental interventions on health remains difficult to study [5]. Several studies have explored whether changes in the amount of faecal indicator bacteria isolated from hands could be used as a measure of compliance with hand washing, or as a proxy marker for a potential health effects of handwashing [6,7]. Other studies have used microbial source tracking to explore transmission pathways of gastro-intestinal pathogens [8,9,10].

Studies have applied different microbiological techniques and indicators ranging from finger imprints on agar plates, environmental swabs, hand swabs, and hand rinses, followed by bacteriological isolation methods of different levels of sophistication [6,11,12,13,14,15,16]. The applied methods require varying degrees of microbiological expertise, budgets and logistics. At the low budget end, Pinfold and colleagues developed a bacteriological indicator of hand washing behaviour by using KF *Streptococcus* agar plates, on which the fingertips of both hands were placed on either half of the plate [7]. The study showed that the number of finger imprints showing *Enterococcus* spp. colonies was negatively associated with hand washing and positively with diarrhoea. At the opposite end, microbial source tracking (MST) studies examining water [8] and hands [9,10] have used sophisticated molecular methods to detect viruses and distinguish human from animal faecal contamination. Simple and cheap tests can be applied to large sample sizes, which increases power to detect differences. Expensive tests can usually only be done in small numbers, although to some extent study power is improved due to the potentially higher specificity to identify specific pathogens.

In the present study we aimed at developing a test that requires no microbiological expertise and no laboratory facilities other than an incubator, and that would be easy to apply in large numbers of people under field conditions with minimal logistical support. We tested the method using three different approaches: In an experimental, longitudinal study we explored the recontamination rate of hands with two faecal indicator bacteria (*Enterococcus* spp. and *E. coli* spp.) after hand washing. Second, we explored whether the test can detect higher levels of bacteria on people’s hands following household practices potentially causing hand contamination. Finally, we explored the association between household characteristics and bacterial contamination.

## 2. Experimental Section

### 2.1. Study Settings and Population**s**

#### 2.1.1. India (Recontamination Study and Cross Sectional Study)

The first part of the study was conducted between July and August 2011 in four villages in the state of Andhra Pradesh, India. This part of rural Andhra Pradesh is a semi-arid land with mainly agricultural activities, pottery and silk farming. Houses are mainly made of brick and cement. Mud huts with thatched roofs are used by some low income families. There is a central water tank in all four villages with a single tap for collection by all the villagers. Sanitation facilities are very scarce. The vast majority of the study population practiced open defection. The longitudinal recontamination study aimed at investigating recontamination after handwashing. It involved 14 mothers or caregivers of children under 5 years of age from two villages. Each of the 14 mothers/caregivers was allocated at random to equal numbers of rounds of: (1) hand washing with soap and (2) no hand washing. Four rounds (2/2) were planned for each mother. We conducted one round per day per mother. Five samples were taken for a hand washing round: prior to hand washing (baseline), immediately after hand washing (time 0), at 0.5 h, 1 h and 1.5 h. For a “no hand washing” round, four samples were taken; baseline, 0.5 h, 1 h and 1.5 h. Only one round per day per person was done. Mothers were asked to wash hands as they would normally do, but with soap (which was not common practice in the study area). Mothers used their own soap for the handwashing (soap was available in all households for other uses). No specific instructions were given to mothers as to how to dry hands (all used their saree) and what to do between rounds.

The Indian cross sectional study served to study the association between individual characteristics (including recent activities) and hand contamination. It enrolled men, mothers of young children and grandmothers in four villages, and included the baseline measurement of the women from the recontamination study. Participants were selected as a convenience sample. A single sample was taken from each participant. A brief questionnaire was used to document the activities done within the half hour prior to taking the sample.

#### 2.1.2. Mozambique (Cross Sectional Study)

The second part of the study took place in six low income settlements (bairros) in Maputo, Mozambique between October 2012 and February 2013, aiming at exploring associations between household-level risk factors and hand contamination. The microbiological survey was done in the context of a baseline study to evaluate the effect of a large urban water and sanitation intervention on diarrhoea in three of the six neighbourhoods (the follow-up study is planned for 2016). At the time of the study, the sanitation conditions were predominantly poorly maintained, unimproved pit latrines with frequent leakage. Open defecation occurred but was uncommon among the study population. A minority used plastic bags (“flying toilets”). Water access to public or private taps was widespread. The microbiological samples were taken from the respondent (usually a female care giver of children) of a household who also answered a brief questionnaire on water, sanitation and health.

### 2.2. Microbiological Sampling and Processing

In all study components, each participant was asked to follow the same technique. The plate was placed on a steady surface. Participants were asked to put all 4 fingertips, excluding the thumb, in a nearly horizontal angle for 2 s onto the agar plate. This was followed by placing the thumb onto the same side of the plate as in the remaining space in the centre of the plate. The fingertips of the other hand were put onto the opposite half of the plate in the same way. Each agar plate was transported from the field in a styrofoam cooler maintaining a low temperature with gel ice packs. In the laboratory the plates were placed in the incubator at 37 degrees Celsius for 24 h.

The samples were tested for *E. coli* and *Enterococcus*, because these are thought to indicate faecal origin of finger contamination, and were therefore used in previous work [7,17,18,19,20,21]. To minimise the laboratory work and the need for microbiological expertise we used the CPS3 chromogenic agar (BioMerieux, Marcy l’Etoile, France) which allows identification of *E. coli* and *Enterococcus* spp. based on the colour and shape of the colonies. This agar also allows identification of *Klebsiella*, *Enterobacter*, *Serratia*, *and Citrobacter*, but these were not included in our analysis despite potentially being of faecal origin. Photo archiving was done for each plate after a 24 h period of incubation. Reading of the plates was done blinded to intervention status by one person (C.D.) in random order. For each plate, the number of contaminated fingers and the overall colony count (all fingertip impressions combined) were separately noted for *Enterococcus* species and *E. coli. Enterococcus* spp. were identified on the CPS3 agar by their small, deep turquoise colonies with sharp borders. A colony was identified as *E. coli* if it was wine red [22].

### 2.3. Statistical Analysis

**Figure 1:**
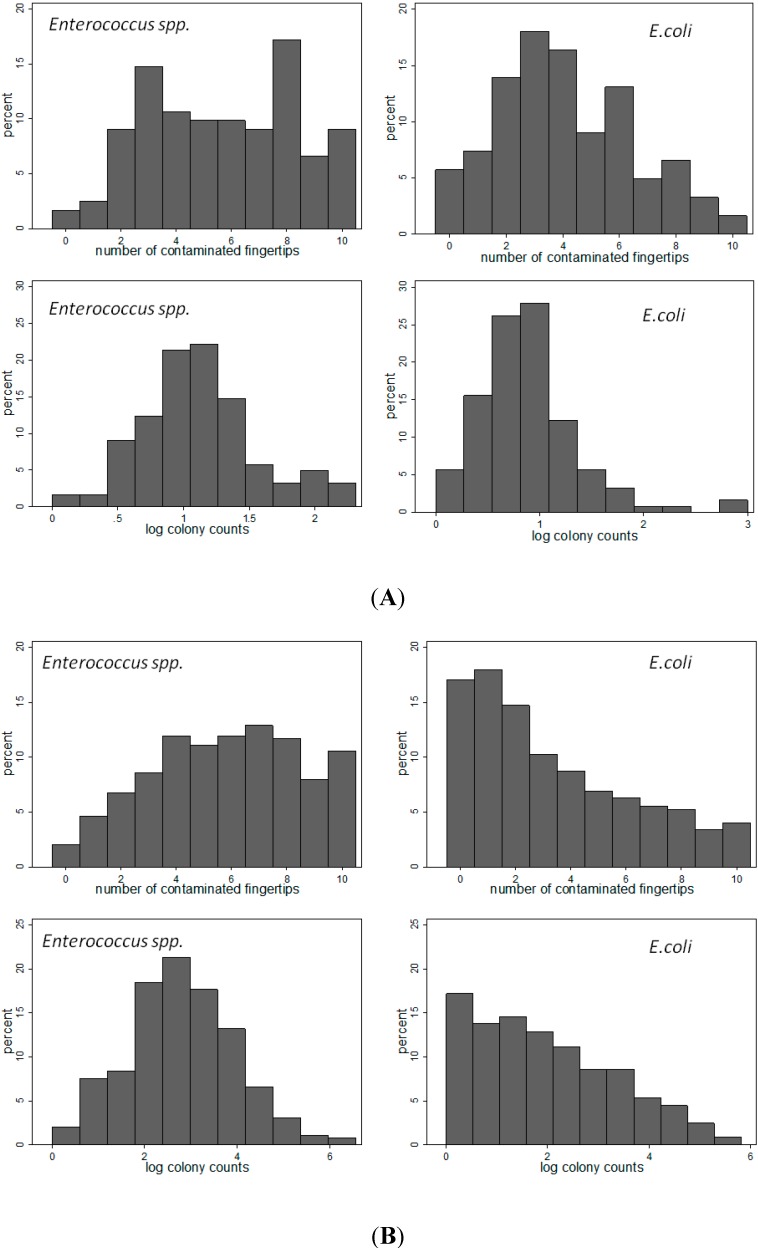
Distribution of number of contaminated fingers and log 10 colony counts. (**A**) cross sectional study India (*N* = 122) (**B**) cross sectional study Mozambique (*N* = 650).

In the recontamination study, we used linear regression analysis to compare the hand washing and no hand washing group with respect to the count of the number of contaminated fingers and number of colonies. To adjust for repeated measurement, person was specified as a random effect. In contrast to the distribution of the number of contaminated fingers, the colony counts were highly right skewed and therefore log-transformed (log 10, Figure 1). A value of 1.0 was added to all colony counts to remove zero counts prior to log transformation. The analysis was adjusted for the baseline contamination level of each woman at each round.

For the two cross-sectional studies in India and Mozambique we used univariate and multivariate linear regression analysis to explore the effect of various activities and potential risk factors for faecal contamination of fingers.

## 3. Results and Discussion

The distributions of finger counts and log colony counts of *Enterococcus* and *E. coli* in India and Mozambique are shown in Figure 1A,B. Log 10 transformation resulted in a near symmetrical distribution of the colony counts, except for *E. coli* spp. in Mozambique.

### 3.1. Longitudinal Recontamination Study

**Figure 2:**
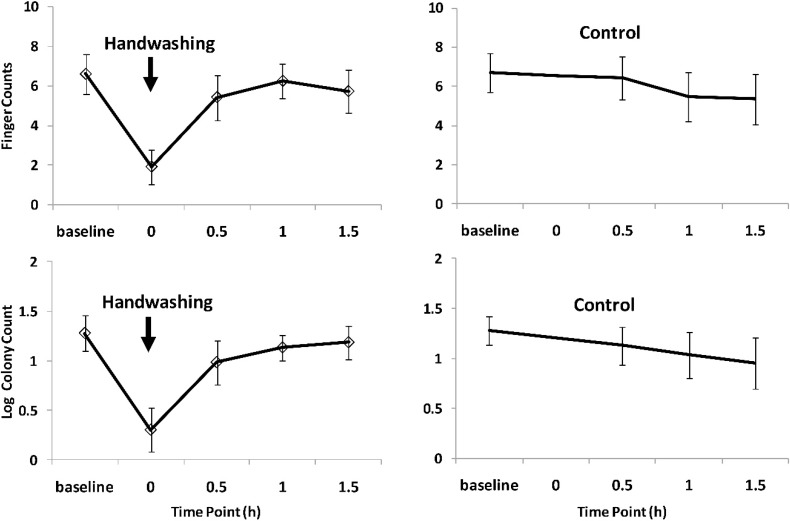
Effect of handwashing with soap on *Enterococcus* contamination of finger tips.

Due to varying availability, only 9 mothers underwent all four rounds (2 hand washing, 2 no hand washing) while 5 mothers had two rounds (one hand wash, one no hand wash), resulting in a total of 23 handwashing and 23 control rounds. Figure 2 shows the mean number of fingers contaminated with *Enterococcus* spp. and the mean log colony count at the different points in time in relation to hand washing. The fastest increase in finger contamination happened between time 0 and 0.5 h after hand washing. One hour after handwashing, contamination appeared to return to the baseline level. The control group data for *Enterococcus* spp. suggest a small decline in contamination with time, both for the mean number of fingers (from 6.7 to 5.3) and the mean log colony counts (from 1.3 to 1.0).

**Figure 3:**
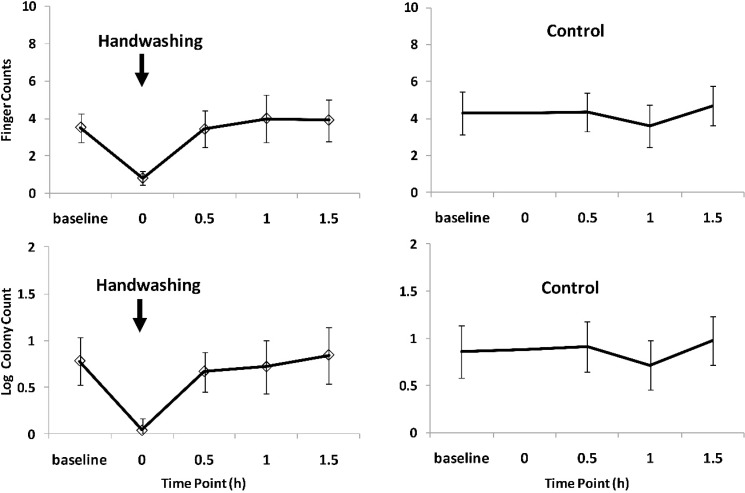
Effect of handwashing with soap on *E. coli* contamination of finger tips.

The analysis of the *E. coli* plate readings showed similar results as the *Enterococcus* spp. (Figure 3). The drop in finger and log colony counts after hand washing was followed by a rapid increase within one hour. The control arm counts revealed no particular trends in *E. coli* contamination over time. Table 1 summarizes the statistical analysis of contamination levels between hand washing and control rounds at each time point. At time 0.5 h contamination in the hand washing arm was consistently lower compared to the control arm for both bacteria (finger and log colony counts), although the *p*-values indicate no statistical support for the difference. At 1 h and 1.5 h there were no indication of a difference between hand washing and no hand washing.

**Table 1:** Longitudinal recontamination study: comparison between the hand washing and control at each time point (for each model *N* = 46 person-rounds in 14 mothers).

Time Point	Difference in Mean Finger Counts and Mean Log Colony Counts between Hand Washing and Control ( *p*-Value) ^*^			
	*Enterococcus* FC	*Enterococcus* LogCC	*E. coli* FC	*E. coli* LogCC
Baseline	−0.87 (0.89)	−0.002 (0.98)	−0.78 (0.11)	−0.08 (0.63)
0.5 h	−0.96 (0.19)	−0.15 (0.26)	−0.72 (0.32)	−0.22 (0.19)
1 h	0.80 (0.29)	0.10 (0.43)	0.52 (0.54)	0.02 (0.92)
1.5 h	0.43 (0.59)	0.23 (0.10)	−0.52 (0.49)	−0.11 (0.57)

FC—finger counts, LogCC—log colony counts; ^*^ linear regression analysis adjusted for baseline values and repeated measurements of participants.

### 3.2. Cross Sectional Study India

Table 2 shows the effect of different activities and the type of person on the contamination with *Enterococcus* and *E. coli*. For *Enterococcus*, child rearing and food preparation were associated with higher finger and log colony counts. There was evidence that mothers of a child under 5 years had a higher number of fingers contaminated and a higher log colony count as compared to grandmothers and males.

In multivariate analysis adding child rearing to the model, the effect of women on levels of contamination with *Enterococcus* changed from 1.64 (0.011) to 1.1 (0.097) with a confidence interval crossing 1 (−0.20 to 2.41). This shows that the effect of female gender on contamination is explained partly by the close relationship of women with activities related to child rearing.

**Table 2:** Cross sectional study India: effect of type of person and type of activity on number of fingers contaminated and log colony count of *Enterococcus* and *E. coli* (*N* = 122).

Person/Activity	*N*	No. of Fingers Contaminated			Log Colony Count		
		Difference ^*^	95% CI	P	Difference ^**^	95% CI	P
*Enterococcus* spp.							
Person							
Male (reference)	23	-		-	-		-
Mother of young child	65	1.45	0.11/2.74	0.033	0.26	0.04/0.51	0.036
Grandmother	34	0.34	−1.15/1.91	0.654	0.11	−0.18/0.35	0.383
Activity							
None (reference)	19	-	-	-	-		-
Child rearing	37	2.35	0.87/3.83	0.002	0.28	0.02/0.52	0.031
Food preparation	12	2.41	1.07/3.74	0.000	0.54	0.17/0.92	0.004
Soil contact	24	0.77	−0.62/2.15	0.276	0.06	−0.18/0.30	0.645
Contact with Agricultural products/crops	6	1.36	−0.21/2.92	0.090	0.24	−0.24/0.71	0.325
Animal contact	10	1.36	−0.52/3.25	0.156	0.17	−0.24/0.58	0.409
Other	23	0.64	−1.19/2.46	0.495	0.10	−0.23/0.43	0.545
*Escherichia coli*							
Person							
Male (reference)	23	-		-	-		-
Mother of young child	65	0.61	−0.48/1.82	0.328	0.08	−0.12/0.37	0.534
Grandmother	34	0.73	−0.66/2.12	0.287	0.19	−0.13/0.49	0.210
Activity							
None (reference)	19	-		-	-		-
Child rearing	37	−0.05	−1.39/1.30	0.947	0.10	−0.22/0.42	0.542
Food preparation	12	1.62	−0.28/3.51	0.095	2.20	1.97/2.42	0.000
Soil contact	24	0.78	−0.59/2.16	0.264	0.14	−0.04/0.32	0.129
Contact with Agricultural products/crops	6	2.83	0.26/5.41	0.031	2.28	2.11/2.45	0.000
Animal contact	10	1.15	−0.56/2.86	0.188	0.27	0.001/0.54	0.050
Other	23	−0.21	−1.86/1.42	0.798	0.03	−0.31/0.38	0.845

^*^ univariate linear regression analysis, difference in mean number of contaminated fingers; ^**^ difference in mean log colony counts.

There was evidence that handling of agricultural produce and crops, food preparation and animal contact were associated with higher *E. coli* log colony and finger counts. There was no evidence that the type of person had a strong impact on the levels of contamination with *E. coli*.

### 3.3. Cross Sectional Study Mozambique

The sample included 650 plates taken from interview respondents. There were marked differences in fingertip/colony counts among the 6 neighbourhoods (Table 3). However, none of the potential risk factors included in the survey, such as type of latrine, water access or education level were strongly associated with fingertip or colony counts of *Enterococcus* and *E. coli.* There was statistical evidence that respondents from higher educated households had lower *Enterococcus* log colony counts, but the size of difference was very small. Respondents living in households reporting a diarrhoea case in the last 7 days did not show higher fingertip or colony counts for either bacteria.

**Table 3:** Cross sectional study Mozambique: Effect of household characteristics on the mean number of fingers contaminated and mean log colony count of *Enterococcus* and *E. coli* (*N* = 650).

Risk Factor	*N*	No. of Fingers Contaminated			Log Colony Count		
		Mean Counts	Difference ^*^	*p* Value ^*^	Mean Counts	Difference ^**^	*p* Value ^*^
*Enterococcus* spp.							
Neighbourhood ID				<0.001			<0.001
1	111	5.7	ref		2.7	ref	
2	157	5.9	0.2		2.8	0.1	
3	92	5.3	−0.4		2.6	−0.2	
4	78	7.1	1.4		3.3	0.5	
5	101	5.0	−0.7		2.4	−0.3	
6	111	6.0	0.4		2.8	0.1	
Latrine type							
Unimproved	158	5.9	ref		2.9	ref	
Improved	492	5.8	−0.1	0.64	2.7	−0.1	0.22
Water tap							
Out of compound	277	5.9	ref		2.8	ref	
In compound	373	5.7	−0.2	0.44	2.8	0.0	0.71
Highest education level in household							
No secondary	338	6.0	ref		2.9	ref	
Some secondary	312	5.6	−0.3	0.11	2.7	−0.2	0.01
Diarrhoea in HH in last 7 days							
Yes	124	5.6	ref		2.7	ref	
No	526	5.8	0.2	0.42	2.8	0.1	0.49
*Escherichia coli*							
Neighbourhood ID				<0.001			<0.001
1	111	4.0	ref		2.2	ref	
2	157	3.4	−0.6		1.9	−0.3	
3	92	2.2	−1.8		1.3	−1.0	
4	78	3.8	−0.2		2.2	0.0	
5	101	3.0	−1.0		1.7	−0.6	
6	111	3.5	−0.6		2.0	−0.3	
Latrine type							
Unimproved	158	3.2	ref		1.8	ref	
Improved	492	3.4	0.2	0.49	1.9	0.2	0.19
Water tap							
Out of compound	277	3.4	ref		1.9	ref	
In compound	373	3.3	−0.1	0.63	1.9	0.0	0.93
Highest education level in household							
No secondary	338	3.4	ref		1.9	ref	
Some secondary	312	3.3	−0.1	0.75	1.9	−0.1	0.61
Diarrhoea in HH in last 7 days							
Yes	124	3.4	ref		1.9	ref	
No	526	3.4	0	0.99	1.9	0.1	0.67

^*^ univariate linear regression analysis, difference in mean number of contaminated fingers; ^**^ difference in mean log colony counts.

### 3.4. Discussion

Using a simple microbiological test for faecal contamination of hands we found that recontamination with *Enterococcus* and *E. coli* following handwashing happens quickly, with baseline levels being reached within one hour. We further found that contamination with *Enterococcus* was associated with mother/child contact, whereas *E. coli* was associated with agricultural practices. In a large scale cross sectional survey in an urban low income setting in Mozambique, there were marked differences in counts among neighbourhoods. However, none of the basic household characteristics such as education, water and sanitation offered an explanation for these differences.

We first tested our method in a low income agricultural setting with poor sanitation and omnipresence of livestock, an environment likely to be highly contaminated with gastro-intestinal pathogens [21]. In such settings, evaluating compliance with hygiene practices such as hand washing using microbial contamination of hands is unlikely to be easy, given that rates of recontamination appear to be very high, as has also been shown in studies using other microbiological methods [20,23]. The ability of microbiological indicators to distinguish people who wash hands from those that do not may be better in less contaminated settings, where recontamination rates appear to be lower [18,19].

In environments that are highly contaminated with pathogens of faecal origin, for example due to an absence of water and sanitation, rapid recontamination of hands may generally limit the potential health benefits of hand washing. However, our knowledge on the relative importance of different transmission pathways (e.g., food, water, environmental, direct human-to-human contact, animals) is currently insufficient to make this conclusion, as most studies (including ours) measured faecal indicator bacteria rather than the pathogens thought to be responsible for most diarrhoea cases in children [24]. Further, most faecal indicator bacteria are not specific for human faecal contamination, unless sophisticated laboratory methods including DNA analyses are applied [8]. Observational studies have shown an association between hand contamination and the incidence of diarrhoea in children [7,25,26,27], while other studies found no association [6]. The results from these studies are likely to be subject to strong confounding by socio-economic status and other factors associated with hand contamination and diarrhoea.

The risk factor analysis in India showed that activities involving contact with crops or agricultural products for example during food preparation increase contamination with *E. coli*, hinting at a transmission path by which different *E. coli* strains may enter households. Current hand hygiene campaigns often emphasise the need for hand hygiene prior to food preparation whereas our results and similar findings from Tanzania [20] suggest that hand contamination could be particularly high after food preparation. These findings could contribute to the ongoing debate on the most important occasions for practicing hand washing to reduce diarrhoea [27]. In contrast to the association between contamination and risk practices found in India, none of the household characteristics explored in the cross sectional study in Mozambique was associated with hand contamination. A potentially strong temporal variation of contamination may lower the ability of a test to explore the effects of water and sanitation on hand contamination. The marked differences in contamination among different neighbourhood may deserve further study. All neighbourhoods were sampled concurrently, *i.e.*, temporally varying factors such as temperature and humidity do not explain the differences. This observation seems to confirm the assumption that neighbourhood level effects dominate transmission of gastro-intestinal pathogens and that an individual household may not gain a health benefit from installing a better latrine or water access unless neighbours do the same [28,29]. However, in our sample, neighbourhood coverage with water and sanitation was not associated with neighbourhood level contamination.

This study is limited by the use of a simple microbiological method, focusing on two bacteria (*E. coli* and *Enterococcus*) that are regarded as indicators of faecal contamination. Both bacteria are not specific to human faeces and can be found in many warm-blooded animals including cows and goats that were common in the India site (but not Maputo). The decrease in *Enterococcus* contamination in the control group over time observed in the recontamination study (Figure 2) may suggest that the process of taking a sample already reduces contamination, and that taking the same number of samples as in the handwashing group would have been preferable. However, the reductions over time were small. Taking an additional sample probably would have made little difference. Other limitations include a small sample size for the various activities in the cross sectional survey and the sampling technique being a convenience sample. Further, as the CPS3 agar is currently mainly used in hospital settings, its accuracy under field conditions needs further confirmation. For training purposes prior to reading of the plates, we conducted limited microbiological confirmation in India by randomly selecting 15 plates from the overall sample of 297 for further testing at the Christian Medical College, Vellore, India, using standard microbiological essays [22]. While these tests confirmed that under field conditions *Enterococcus* and *E. coli* display the expected morphology on CPS3 agars [22], a larger sample and independent species identification will be required to test the accuracy of CPS3 in the field.

In Maputo, the plates and incubator were delivered to a data collection agency with user instructions given by phone and email, which highlights the simplicity of the test method. Apart from an incubator and a digital camera, the method requires the availability of the chromogenic agar plate (price per plate around 1.5 EUR). Another attractive feature of the test may be the near-normal distribution of the number of contaminated fingers and the log-transformed colony counts, which facilitates the statistical analysis (other bacterial count data are often highly skewed or zero-inflated even after transformation). Some hand contamination studies use rinsing of the whole hand in sterile water in a plastic bag [20]. We only assessed contamination of fingertips which however may contribute most to transmission of pathogens.

## 4. Conclusions

To conclude, the ability of the test to evaluate specifically compliance with hand hygiene may be limited by the quick recontamination of hands in highly contaminated environments. The test reflected risk practices preceding taking of the sample. The test also appears to be suitable to distinguish levels of hand contamination among neighbourhoods, but was unable to identify reasons for these differences. This research was made possible with funding from the SHARE Research Consortium and the UK Department for International Development (DFID) however the views expressed do not necessarily represent the department’s policy. The views expressed are those of the authors.
